# An Unusual Case of Mandibular Squamous Cell Carcinoma in Intimacy with an Impacted Wisdom Tooth

**DOI:** 10.1155/2019/8360357

**Published:** 2019-04-11

**Authors:** Johnson Cheung, Ayham Al Afif, Martin Joseph Bullock, Chad Robertson, Robert Hart, Reginald Goodday

**Affiliations:** ^1^Department of Oral and Maxillofacial Surgery, Dalhousie University, 5981 University Avenue, Room 5132, P.O. Box 15000, Halifax, Nova Scotia, Canada B3H 4R2; ^2^Division of Otolaryngology-Head and Neck Surgery, Dalhousie University, QEII Health Sciences Centre 3rd floor Dickson Building, 5820 University Avenue, Halifax, Nova Scotia, Canada B3H 1Y9; ^3^Department of Pathology, Dalhousie University, Sir Charles Tupper Medical Building, Room 11B, 5850 College Street, PO Box 15000, Halifax, Nova Scotia, Canada B3H 4R2

## Abstract

Squamous cell carcinoma is the most common head and neck malignancy. It can occur in the mandible or maxilla without a preexisting oral mucosal lesion. Often, the clinical and radiographic presentation of SCC directs the clinician to favour malignancy over other pathological conditions. However, SCC may also mimic an infectious condition and therefore can pose a diagnostic challenge even for the most experienced clinicians. Herein, we report a case of mandibular squamous cell carcinoma in a 53-year-old male who presented with symptoms of right facial swelling, trismus, pain, and right-sided lip paresthesia. The patient underwent a surgical removal of the presumed infected third molar of the right mandible, but histopathological analysis of the associated soft tissue unexpectedly yielded squamous cell carcinoma. Given the biopsy-proven diagnosis, the patient received a mandibular resection of the tumor followed by primary reconstruction with a fibular free flap. Patients presenting with symptoms mimicking odontogenic infections should receive vigilant attention by clinicians with regard to the disease history, clinical signs, radiographic evidence, and decision for histopathological analysis. This is especially true in the context of impacted dentition, where malignancy must be considered when formulating a differential diagnosis.

## 1. Introduction

Squamous cell carcinoma (SCC) is the most common cancer of the oral cavity and accounts for up to 95% of all cancers in this anatomical region. Most commonly, it occurs on the tongue (40% of cases) and the floor of the mouth (30%). Other sites include the buccal mucosa, gingiva, palate, and retromolar region [1–3]. Males are affected more than females (2 : 1), especially at an age greater than 50 years [1, 4]. The prognosis for oral SCC is guarded but is also dependent on the site of the primary lesion. Five-year survival rates of 20% (floor of the mouth) and up to 60% (gingival) have been reported [1, 3]. Nodal metastasis is a known poor prognostic indicator [5]. Recurrence rates of up to 70% have been reported for mandibular SCC [6].

Oral SCC most commonly presents as an indurated, ulcerated mucosal lesion [7, 8]. However, occasionally, it may be challenging to distinguish SCC from more common maladies such as an odontogenic infection. An example of such is when the tumor exists in intimacy with an impacted tooth. Therefore, keen clinical and radiographic inspection along with a tissue biopsy is paramount, as it may reveal more ominous pathology.

Herein, we report a case of mandibular SCC, which developed in close association with an impacted wisdom tooth, clinically mimicking an odontogenic infection. We present the case details, histopathological findings, and management. We also discuss recommendations for the management of impacted wisdom teeth, especially in the context of associated pathology or disease. Through this report, we aim to highlight the importance of being vigilant for such lesions, as to avoid diagnostic pitfalls. Malignancy must always be on the differential diagnosis when dealing with pathology involving impacted third molars.

## 2. Case Presentation

### 2.1. Clinical Presentation

A 53-year-old male presented to our centre with a one-month history of worsening right mandibular swelling, pain, and progressive trismus. He had been diagnosed with an odontogenic infection by emergency physicians and was treated in the emergency room with 2 g of intravenous (IV) cefazolin daily over four days without relief before being ultimately referred to our care.

The patient's past medical history was remarkable for a benign Rathke's cleft cyst involving the pituitary gland, which was previously resected. His medications included hydrocortisone, levothyroxine, and testosterone as hormone replacement, as well as codeine on an as-needed basis for migraines. He was otherwise healthy, with no allergies. He reported a 20-pack year smoking history, as well as occasional, but infrequent, alcohol and marijuana use.

**Figure 1:**
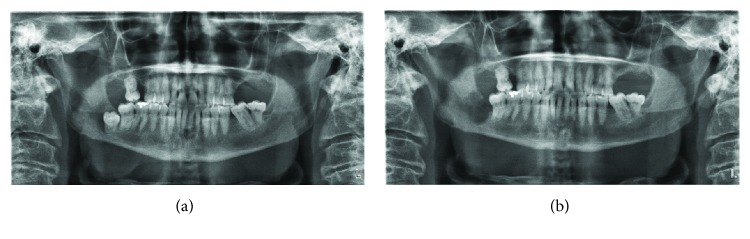
(a) Orthopantomogram of the presenting case illustrating the ill-defined radiolucent lesion associated with impacted right mandibular third molar. (b) Immediate postoperative imaging showing postextraction of the right mandibular third molar and biopsy of the right mandible. Note that the mandibular inferior border remained intact.

**Figure 2:**
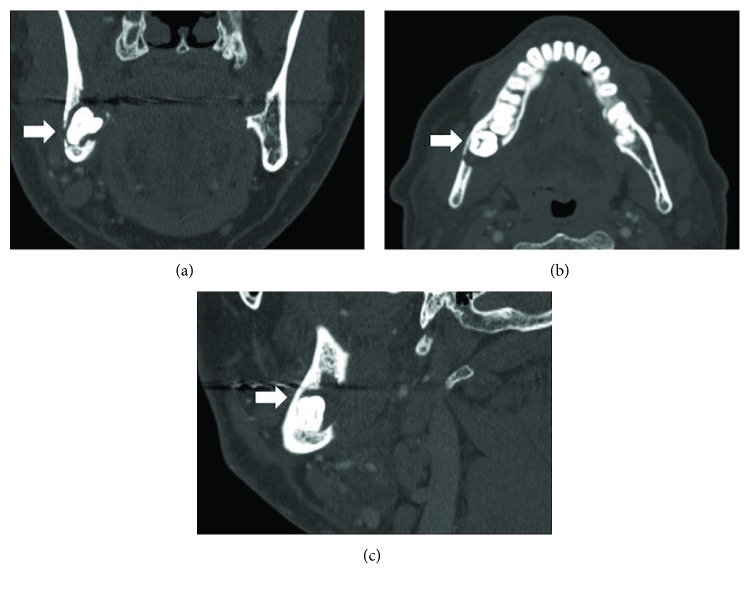
Coronal (a), axial (b), and sagittal (c) views on CT scan of facial bones illustrating the impacted right mandibular third molar with the associated radiolucent lesion and its relationship to the surrounding remaining mandibular bone. Note the cystic appearance of the lesion. White arrow in each panel indicates the impacted right mandibular third molar in association with the radiolucent lesion.

On presentation, he was in no distress and was afebrile with stable vital signs. Clinical examination revealed mild, firm, and nonfluctuant swelling of his right posterior mandible with no overlying skin changes. Palpation elicited diffuse tenderness along the right submandibular region but no palpable cervical lymphadenopathy was appreciated. Cranial nerve exam revealed paresthesia and loss of sensation along his right lower lip. No other cranial nerve deficits were noted. There was marked trismus with a maximum incisal opening of 16 mm. Intraoral examination revealed a healthy dentition with no obvious decay. There was some minor erythema of the mucosa distal to the right mandibular second molar, which was tender to palpation. Otherwise, there were no obvious mucosal changes. Flexible nasopharyngoscopy showed no abnormalities in the pharynx. The remainder of neurological, cardiovascular, abdominal, and musculoskeletal exams was unremarkable. Imaging of the patient's facial bones via orthopantomogram and computed tomography (CT) was also obtained (Figures 1 and 2).

### 2.2. Orthopantomogram

A vertically impacted right mandibular third molar was present in the posterior right mandible, with an associated ill-defined radiolucency (Figure 1). This had a “moth-eaten” appearance, which extended posteriorly to involve the mandibular ramus. The lesion obscured the continuity of the mandibular canal. There was no pathological fracture. The lamina dura of the posterior dentition was poorly defined with no root resorption of the posterior dentition, although the right mandibular second molar appeared to be tipped slightly distally. No bony expansion or periosteal reaction of the inferior border could be detected. Caries was also incidentally noted on the distal of the right mandibular second molar.

### 2.3. CT of Facial Bones and Neck

CT imaging revealed destruction of the right posterior mandible by the radiolucent lesion described above, which measured 12 × 7.3 mm in dimension (Figure 2). The right mandibular third molar remains deeply impacted in the mandibular angle/ramus region. The osteolytic lesion had eroded through the lingual cortical plate and also involved the buccal cortex as well. The inferior alveolar nerve canal could still be visualized but was in very close proximity to the lingual aspect of the right mandibular third molar. There was evidence of surrounding soft tissue reaction and inflammatory changes. There were several subcentimeter, nonnecrotic right perifacial and jugulodigastric nodes (levels IB, IIA). The presence of a focal abscess could not be ruled out on CT imaging alone.

Given the patient's clinical and radiographic presentation, our working diagnosis was an odontogenic infection secondary to impacted right mandibular third molar. Therefore, the decision was made to treat the presenting condition as an odontogenic infection by performing an incision and drainage under general anesthetic. In light of the progression of the patient's symptoms over the previous four days despite cefazolin therapy, it was felt that antibiotics alone would not be sufficient treatment.

### 2.4. Initial Biopsy

Following the administration of a general anesthetic, a distal-releasing incision of the mucosa overlying the right mandibular second molar was made, followed by elevation of a full thickness mucoperiosteal flap. No mucosal ulcerations were detected along the right posterior mandible. The impacted right mandibular third molar was removed without complications. This was successful only after the removal of abundant surrounding soft tissue which had a gelatinous texture. We also noted very minimal soft or bony tissue hemorrhage intraoperatively. The mesial and inferior mandibular walls of the third molar socket were deemed to be intact. The lingual wall was thin and barely intact, and the distal wall was poorly visualized owing to the large concavity of the socket. Approximately 1.5 cm of aggregate soft tissue along with the extracted right mandibular third molar was submitted for histopathological and microbiological analysis. Although it was near the surgical bed, the inferior alveolar nerve was identified and preserved.

**Figure 3:**
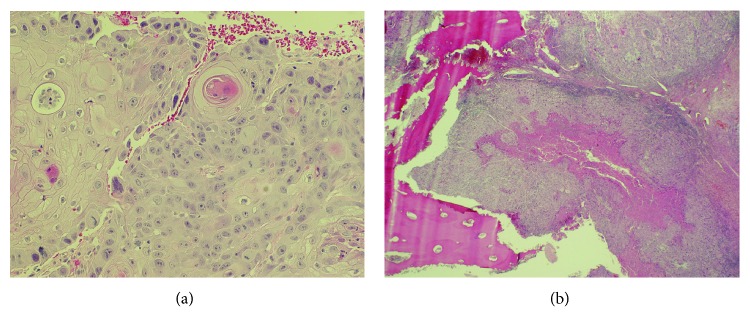
Histopathology from initial biopsy of lesion and final resected specimen. (a) High-power (200x, H&E stain) magnification illustrating fragments of keratinizing conventional SCC from incisional biopsy of lesion. (b) Low-power (40x, H&E stain) magnification of tissue from final resected specimen showing SCC invasion into mandibular bone (lower left) with central tumor necrosis.

Histopathological analysis showed multiple fragments of soft tissue that were nearly completely replaced by moderately differentiated invasive keratinizing squamous cell carcinoma (Figures 3(a) and Supplemental Figure 1A). A molar tooth was also submitted which was not examined microscopically. The patient was informed of the diagnosis on follow-up, and CT imaging of the thorax showed no evidence of metastatic disease in the thoracic cavity. Therefore, staging of the tumor was determined to be T4A N0 M0. The hospital's head and neck tumor board recommended that he undergo oromandibular resection, selective neck dissection, and fibular free flap reconstruction for his SCC, two weeks from the time of diagnosis. He was also planned for adjuvant radiation therapy.

### 2.5. Resection, Reconstruction, and Perioperative Care

**Figure 4:**
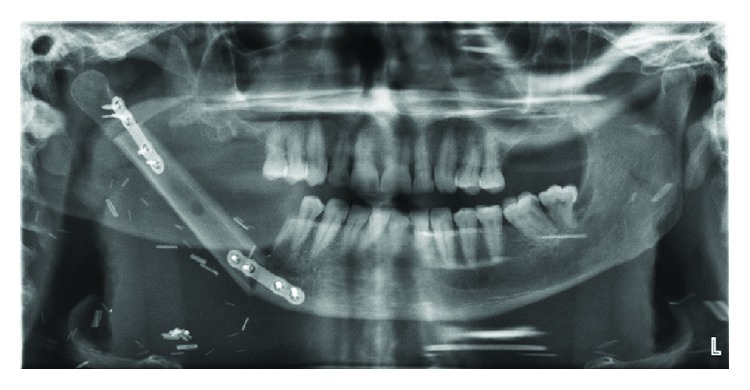
Orthopantomogram illustrating the fibular bony segment of the free flap, plated to the adjacent native mandible as part of the reconstruction. This was obtained on postoperative day 7.

After consent was obtained, the patient was brought to the operating theatre for a tracheostomy, oromandibular resection, selective neck dissection, and right fibula free flap reconstruction. Following induction, he underwent a successful orotracheal intubation, which was followed by tracheostomy in standard fashion. We planned a half apron incision, with a vertical midline limb to facilitate a lip split; care was taken to avoid communication with the tracheostomy incision. Following the incision and subplatysmal flaps, level IA/B neck dissection was commenced. Nodes in this compartment were adherent to the inferior border of the mandible, involving the marginal branch of the facial nerve, which was sacrificed. The right mandibular canine was extracted, defining the anterior limit of the mandibulectomy, performed using a reciprocating saw. The tumor was infiltrating the parotid gland laterally. The lingual nerve was involved inferomedially, necessitating sacrifice of the lingual nerve. Soft tissue along the floor of the mouth extending to the retromolar trigone and palatoglossus was included in the resection. The posterior mandibulectomy was completed 1 cm away from the tumor. The specimen was removed en bloc, followed by a selective neck dissection of levels II-III. Frozen sections obtained were all negative. The defect was reconstructed with a right fibula free flap, with a 6 × 8 cm laterally-based skin paddle (Figure 4). Vascular anastomoses were secured to the right facial artery and left external jugular vein, and an implantable arterial Doppler was placed. The flap donor site was closed with a split thickness skin graft. The patient was allowed to slowly awaken from the anesthetic. He was fed on the 7th postoperative day, and the Doppler was removed on the 9^th^ postoperative day. His hospital stay was uncomplicated and his flap was healthy on discharge.

### 2.6. Histopathology of Final Resected Specimen

The main resection specimen was a right oromandibular resection with level IA/B neck dissection and partial parotidectomy. There was a 1.5 cm eroded mucosal defect in the retromolar area that corresponded to the site of the biopsy. A 3 cm mass extended from this defect into the underlying bone. The tumor was predominantly intraosseous but also involved the surrounding soft tissue. Microscopy confirmed an invasive squamous cell carcinoma that extended from the eroded defect into the bone (Figures 3(b) and Supplemental Figure 1B). Focal mild dysplasia was noted in the retromolar mucosa, suggesting an origin from this area rather than an intraosseous origin. The bone, soft tissue, and mucosal margins were all free of malignancy, as were 23 levels I-III lymph nodes. Microbiology results from culture and sensitivity testing showed no growth of any microorganisms after 48 hours of incubation.

## 3. Discussion

We present a case of a gentleman with signs, symptoms, and radiographic findings compatible with an odontogenic infection related to an impacted wisdom tooth. However, upon further analysis, the patient's condition proved to be T4a N0 M0 SCC of the oral cavity. It is tempting in such cases to treat the patient by removing the offending tooth, incising and draining any associated abscess, and treating with antibiotics. However, clinicians should be very cautious of hastily removing a tooth associated with underlying malignancy without further workup, since doing so has been linked to poorer patient prognosis [9]. This was illustrated by Naruse et al. [9] for which at least 3 patients underwent initial tooth extraction before being diagnosed with SCC 1 to 2 months later in the same anatomical location. If the tooth is to be removed, we recommend obtaining a sample of any associated tissue for histopathological analysis, especially when clinical history, radiographic presentation, and intraoperative findings are atypical for an odontogenic infection.

Several conditions should be on the clinician's differential diagnosis in such cases. Benign lesions include osteomyelitis, medication- or radiation-associated osteonecrosis, dentigerous cyst, ameloblastoma, odontogenic keratocyst, and central giant cell granuloma. Malignant possibilities include but are not limited to SCC, malignant odontogenic tumors (such as ameloblastic carcinoma), salivary gland malignancies (such as intraosseous mucoepidermoid carcinoma), osteosarcoma, and metastatic disease to the jaws. Moreover, osteomyelitis was favoured in the differential over malignancy given the moth-eaten appearance of the orthopantomogram that resembled sequestra (Figure 1), which is now in fact a representation of the bony destructive process from the tumor. Interestingly, the CT imaging offered a more cystic appearance (and without sequestra) that favoured a more benign process, although the radiolucency may also represent bony destruction as well (Figure 2). Alternative imaging modalities during the initial workup include cone beam CT. An advantage of this modality includes a better resolution of bony osseous lesions allowing for improved preoperative characterization of the extent of disease. Moreover, the use of cone beam CT has demonstrated a higher sensitivity in detecting SCC invasion into the bone than traditional CT, albeit with lower specificity [10]. Other imaging modalities may include an ultrasound of the neck and face, to rule out underlying fluid collections suggestive of a localized abscess [11]. A recent study showed that magnetic resonance imaging (MRI) performed better than CT at initially diagnosing odontogenic infections especially when delineating the soft tissue boundaries [12]. However, easy access to this method may be centre-dependent and also requires longer scan times and increased costs. The unusual clinical appearance and lack of clinically evident mucosal ulcerations did not initially favour the diagnosis of SCC. Notably, other primary osseous malignancies are also not typically associated with mucosal lesions. It has been suggested that although SCC of the retromolar trigone account for only 12% of oral SCCs, the extent of this disease entity can be severely underappreciated [13, 14]. Malignancies of the retromolar trigone usually present late, with alarming symptoms of trismus, neuralgia, otalgia, or trigeminal paresthesia [15]. In the present case, the location of SCC was in the retromolar trigone region, and the patient had at least two of the mentioned symptoms on presentation.

Although there was no clinically visible mucosal lesion at the time of initial biopsy, histopathology and microscopic exam of the resected soft tissues revealed that the tumor originated from the oral epithelium with subsequent invasion into the bone (Figure 3(b)). This pattern of tumorigenesis did not favour the diagnosis of a primary intraosseous SCC, in which the tumor originates from within the bone itself [16]. In our patient, early dysplastic changes of the squamous epithelium on the retromolar mucosal surface were evident on histopathological analysis of the operative specimen, suggesting that the lesion originated from the mucosa, rather than intraosseous. Also, there is no evidence that this tumor arose from the follicular epithelium or cystic lining as one may expect when an impacted wisdom tooth (and its follicular tissue) lies in the vicinity of the cancer [17]. Bone invasion is a commonly reported pattern of disease spread for SCCs involving the retromolar trigone [13], and the incidence of invasion into mandibular bone for all oral SCC types is estimated to range from 12 to 56% [18]. The mechanism of bone invasion by SCC is largely facilitated by the bone-resorptive properties of osteoclasts. The bony microenvironment is enriched with cytokines and growth factors, such as transforming growth factor *β* (TGF-*β*), that enable tumor proliferation and inhibition of apoptosis. Furthermore, oral SCC have been found to produce the cytokine interleukin- (IL-) 6, parathyroid hormone-related protein (PTHrP), prostaglandins E_2_ and F_2_, and receptor activator of nuclear factor kappa-*β* ligand (RANKL) to stimulate osteoclastogenesis. These mediators are thought to aid in the invasion of bone by SCC [19]. In the present case, the patient's significant smoking history (20-pack years) may have also conferred a strong risk factor towards his natural course of disease and diagnosis [1, 20].

Upon further history taking, the patient was unaware that he had an impacted wisdom tooth in his right mandible. He never had any symptoms and never received any notice from a dentist regarding its existence. As a reminder, and according to the American Association of Oral and Maxillofacial Surgeons (AAOMS) Task Force on Third Molar Summary, an asymptomatic wisdom tooth does not always constitute an absence of disease [21].

In light of the findings from this case report, we propose the following clinical recommendations:

- If a presumed odontogenic infection does not respond to appropriate therapy, one must reconsider the diagnosis
- If a patient makes the decision to retain impacted third molars, then they should be made aware of the risks involved and that follow-up on a regular basis should be conducted
- If an isolated periodontal defect is noted on clinical or radiographic exam, we recommend a biopsy of the associated soft and/or hard tissue, rather than presuming the diagnosis of periodontal disease
- Soft tissue associated with an impacted tooth with a surrounding osteolytic defect must be submitted for histopathological analysis and not simply regarded as “inflammatory/granulation tissue or follicle”
- Maintain a broad differential diagnosis for mixed radiolucent lesions of the mandible and maxilla associated with pain and swelling while keeping in mind that malignancy is a possibility

## 4. Conclusions

The clinical presentation of oral SCC can mimic an odontogenic infection. Certain clinical features such as swelling, pain, and trismus can lead the clinician down the path of treating an infectious cause, and the temptation is made stronger in the presence of radiographic features that may favour an infective process. Therefore, misdiagnosis or delayed diagnosis may jeopardize prompt management, hampering patient outcome. As with the present case, an odontogenic infection was the presumed diagnosis but the intraoperative decision to biopsy the soft tissue associated with the impacted wisdom tooth revealed SCC. Thus, practitioners are encouraged to be vigilant when treating impacted teeth that present with a combination of clinical and radiographic features, which may resemble an odontogenic infection (i.e., swelling and paresthesia with osteolysis of surrounding bone). Lastly, associated soft and hard tissues presented in such a context should be submitted for histopathological evaluation to rule out a malignancy.

## Supplementary Materials

Supplementary Materials Supplemental Figure 1: (A) low-power (40x, H&E stain) magnification showing fragments of moderately differentiated SCC from incisional biopsy. (B) High-power (200x, H&E stain) magnification showing moderately differentiated SCC with central keratinization from final resected specimen.
