# Supplementary material for: An Unusual Case of Mandibular Squamous Cell Carcinoma in Intimacy with an Impacted Wisdom Tooth
Source: Case Rep Surg. 2019 Apr 11;2019:8360357. doi: 10.1155/2019/8360357 (PMC6487125; doi:10.1155/2019/8360357)
Supplement: Supplementary Materials — Supplemental Figure 1: (A) low-power (40x, H&E stain) magnification showing fragments of moderately differentiated SCC from incisional biopsy. (B) High-power (200x, H&E stain) magnification showing moderately differentiated SCC with central keratinization from final resected specimen. [file 8360357.f1.pdf]

A)

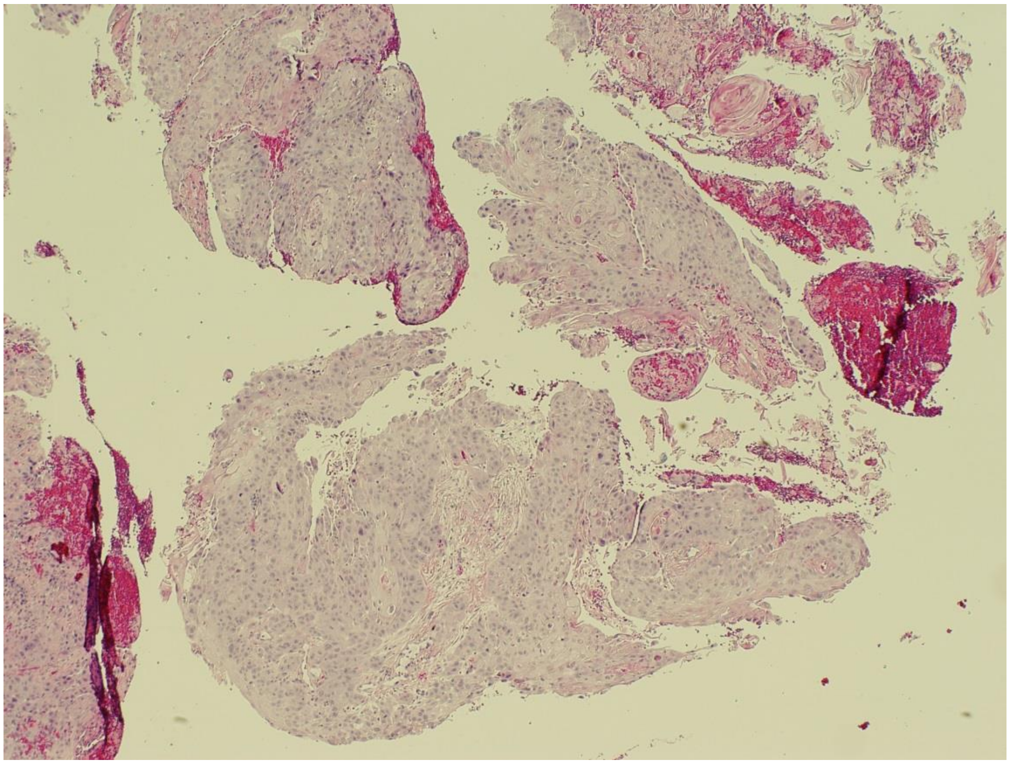

B)

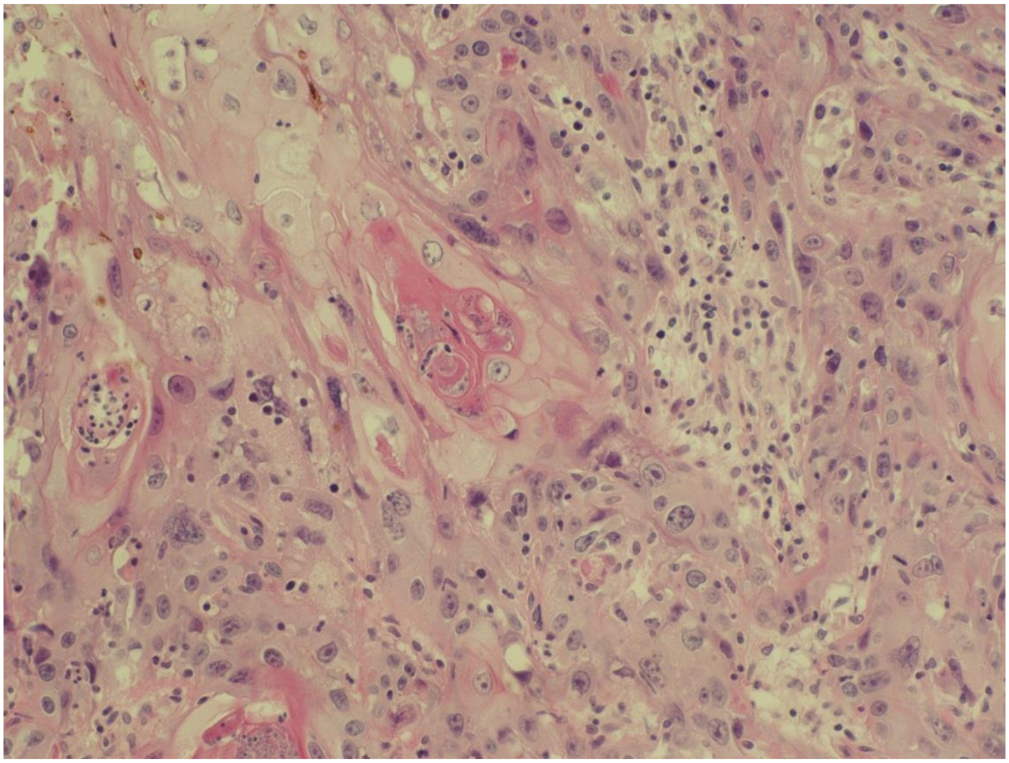

Supplemental Figure 1. **A)** Low power (40x, H&E stain) magnification showing fragments of moderately differentiated SCC from incisional biopsy. **B)** High power (200x, H&E stain) magnification showing moderately differentiated SCC with central keratinization from final resected specimen.
